# Induction of labor after one previous cesarean: Time for the MIBAC trial

**DOI:** 10.1111/aogs.70327

**Published:** 2026-07-22

**Authors:** Sophia Brismar Wendel, Tove Wallström

**Affiliations:** ^1^ Department of Women's and Children's Health Karolinska Institutet Stockholm Sweden; ^2^ Department of Obstetrics and Gynecology Karolinska University Hospital Stockholm Sweden; ^3^ Department of Clinical Science and Education Karolinska Institutet Stockholm Sweden; ^4^ Department of Obstetrics and Gynecology Södersjukhuset Stockholm Sweden

Induction of labor after one previous cesarean has become a common clinical problem. Cesarean delivery is among the most frequently performed major operations in women, and in the Nordic countries, one in five births is by cesarean. Induction now precedes roughly a third of all births, and its indications continue to broaden to include diabetes, preeclampsia, fetal growth restriction or acceleration, prelabor rupture of the membranes, post‐term pregnancy, and more. As a consequence, an increasing number of women reach term with one uterine scar and an indication for delivery and are offered induction of labor rather than repeat cesarean. How best to induce these women is therefore a question of growing clinical relevance.

Labor induction in a scarred uterus differs from spontaneous labor. The principal safety concern is uterine rupture, a rare but potentially catastrophic event. In women with one previous cesarean and spontaneous onset of labor, the risk of uterine rupture is generally below 1%. With induction, it rises to 2%–5% in Nordic cohorts, depending on the method.^1^, ^2^, ^3^

The choice of method is therefore important. Major guidelines from FIGO, the RCOG, and ACOG concur in recommending mechanical cervical ripening, principally the transcervical balloon catheter, and in listing misoprostol as contraindicated after a previous cesarean.^4^, ^5^, ^6^ Notably, the balloon catheter, most often a Foley catheter, is itself generally used off‐label for cervical ripening.

However, the evidence underlying this contraindication is weaker than its near‐universal status suggests. The original concern arose largely from older studies using high doses of vaginal or sequential prostaglandins, in which induction was rarely separable from oxytocin augmentation. In Norwegian population data, the highest risk of complete uterine rupture is associated not with any single agent but with sequential prostaglandin‐then‐oxytocin regimens.^7^ The frequently cited estimate of an approximately 15‐fold increase in rupture with induction derives from a cohort analysis that pooled all prostaglandins rather than isolating any individual agent or dose.^8^

Low‐dose oral misoprostol differs in important respects. Some clinical scenarios favor oral misoprostol over a vaginal method (mechanical or pharmacological), for example, in prelabor rupture of membranes, where a device or repeated vaginal examinations carry a theoretical infection risk, or when the patient declines vaginal examination. Balloon placement specifically is often discouraged when the fetal head is not engaged, given the associated risk of cord prolapse. Moreover, misoprostol's short half‐life allows titration against uterine activity. In the general obstetric population, a 25‐μg oral regimen is at least as effective as vaginal dinoprostone, with fewer cesarean deliveries and substantially less uterine hyperstimulation.^9^ However, the trials establishing this profile excluded women with a uterine scar, so this regimen has essentially not been evaluated in the population in which misoprostol is presumed to be hazardous.^9^

These concerns are not only theoretical. In the largest Swedish series to date, women induced after one previous cesarean with an unfavorable cervix had near‐identical uterine rupture rates with low‐dose oral misoprostol and with a balloon catheter (2.0% vs 2.1%), whereas vaginal dinoprostone was associated with more than double that risk.^3^ Vaginal birth was achieved in close to 70% of cases with both methods. The study was retrospective and confounded by indication, and its authors were among the first to call for the randomized comparison that their data could not provide.^3^

This situation represents genuine clinical equipoise. Misoprostol is withheld in women with a previous cesarean based on evidence from other doses, routes, and agents, whereas the recommended balloon catheter rests largely on observational data, and the only direct comparison available suggests that the two methods may be equivalent. Such equipoise is the appropriate basis for a randomized trial.

The MIBAC trial (Misoprostol or Balloon After Cesarean) is designed to provide this comparison. It is a randomized non‐inferiority trial in which women undergoing induction of labor after one previous cesarean are allocated to low‐dose oral misoprostol, 25 μg, or to a transcervical balloon catheter, the guideline‐preferred method. The primary comparison uses three co‐primary outcomes, tested hierarchically for non‐inferiority: perinatal complication severity and maternal complication severity, each analyzed as an ordinal scale ranging to death, followed by the proportion of vaginal deliveries, for which superiority is also tested if non‐inferiority is met. Uterine rupture is treated as an exploratory outcome, captured within the maternal complication scale, with predefined safety monitoring to detect any signal of harm.

The Nordic countries are well suited to conduct such a trial. They combine high rates of attempted vaginal birth after cesarean, universal maternity care, complete and validated population registers, and an established tradition of pragmatic, register‐based randomized trials. Nordic cohorts have characterized how induction method, oxytocin augmentation, and labor duration relate to uterine rupture in the scarred uterus,^2^ and Finnish and Swedish investigators have compared the balloon catheter with oral misoprostol and studied induction specifically after previous cesarean.^3^, ^10^ The Nordic Federation of Societies of Obstetrics and Gynecology (NFOG) and its clinical trials alliance, NORD‐ACT, provide an established collaborative framework.

Such a trial requires broad participation from the Nordic countries. The required sample size is estimated to be 2500 women. Although the eligible population is substantial, estimated at 1500 women per year in Sweden, no more than approximately 20% of eligible women typically consent to participation in trials of this kind. Recruitment within a single country would therefore extend over several years, during which clinical practice and guidelines might change. A multinational Nordic effort is consequently needed to complete recruitment within a clinically relevant timeframe.

A skeptic might argue that induction after one previous cesarean is too late, but this overlooks why induction remains worthwhile for those who do wish to attempt vaginal birth. Chances of success are substantial: in the largest Swedish series, close to 70% of women achieved vaginal birth regardless of induction method.^3^ Avoiding a further cesarean also reduces the cumulative risks of repeat cesarean, such as placenta previa and accreta spectrum disorders, uterine niche formation, and scar pregnancy in future pregnancies. Few interventions have been shown to reduce cesarean delivery rates; for women who wish to avoid a further cesarean, identifying the safer and more effective induction method is the next‐best opportunity to do so.

Few questions in contemporary obstetrics combine comparable clinical importance, frequency, and uncertainty of direct evidence. A contraindication based largely on outdated and indirect data warrants formal evaluation rather than continued assumption, and women facing this decision should be offered a genuine comparison of the available methods. The Nordic obstetric community has the healthcare systems, collaborative structures, and clinical equipoise required to provide it. The MIBAC trial is timely, and its success will depend on broad participation across the Nordic countries.

## FUNDING INFORMATION

This study was funded by the Healthcare Region of Stockholm (ALF Medicine grant number 985786).

## CONFLICT OF INTEREST STATEMENT

SBW and TW are two of the investigators in the MIBAC trial. SBW received an honorarium in May 2026 from Norgine for a lecture on induction data and induction methods in Sweden.

## Data Availability

Data sharing not applicable to this article as no datasets were generated or analysed during the current study.
